# Cannabidiol for Toothache: Ups, Downs, and Regulatory Considerations

**DOI:** 10.1177/00220345231223691

**Published:** 2024-02-12

**Authors:** K.N. Theken, E.V. Hersh

**Affiliations:** 1 Department of Oral Surgery and Pharmacology, University of Pennsylvania School of Dental Medicine, Philadelphia, PA, USA

**Keywords:** pulpitis, pain, pharmacology, drug interactions, off-label use, decision-making

Toothache is a frequent cause of emergency department visits for dental complaints (Lewis et al. 2015). Because emergency departments are not equipped to provide definitive dental care, analgesic medications, including nonsteroidal anti-inflammatory drugs (NSAIDs), acetaminophen combined with opioids, and topical local anesthetics, are frequently prescribed. However, most clinical trials for acute dental pain have been performed in patients undergoing third molar extraction, as the Food and Drug Administration (FDA) considers dental impaction pain a pivotal surgical model for drugs to receive approval for acute pain (Hersh et al. 2020). In contrast, fewer studies have been performed in patients presenting with spontaneous toothache pain, and several logistical challenges are present compared to postoperative dental pain studies: patients with spontaneous toothache pain cannot be scheduled in advance, toothache patients are often at the very highest end of pain intensity at presentation, and the length of the observation period is limited due to the necessity that definitive dental care (endodontic therapy or exodontia) be performed that day. Consequently, the only placebo-controlled trials in toothache pain patients performed to date have studied the safety and efficacy of topical benzocaine (Hersh et al. 2005, 2013). While recently published guidelines for the treatment of acute dental pain recommend ibuprofen (alone or in combination with acetaminophen) and naproxen sodium as first-line drugs, the conclusions for toothache pain for systemic analgesics were all extrapolated from impacted third molar surgery pain data (Carrasco-Labra et al. 2023). In this issue of the *Journal of Dental Research*, Chrepa et al. (2023) report the results of a phase 2A study investigating the analgesic effects of cannabidiol (CBD) in toothache pain. This study represents the first placebo-controlled trial of a systemic analgesic in toothache pain and is an important proof of concept for a novel pain management strategy in this patient population.

Interestingly, Chrepa et al. (2023) reported a relatively large placebo response, with 25% to 50% of patients reporting pain reduction at various data collection time points. This is consistent with our own observations with topical 20% benzocaine, where the polyethylene glycol vehicle produced a response rate of 47% and benzocaine a response rate of 87% (Hersh et al. 2005). In contrast, placebo response rates in dental impaction studies are typically only 15% to 25% (Hersh et al. 2004; Theken et al. 2019). The “on-and-off” nature of toothache pain in many patients may partially explain this difference.

Although CBD decreased baseline pain within 15 to 30 min, the onset of effect compared to the placebo solution was slow, as the pain scores in the CBD-treated patients did not significantly differ from placebo until 90 to 120 min after administration. Topical 20% benzocaine, compared to its polyethylene glycol vehicle, has an onset to effect of 5 min but a duration of activity of only 30 min (Hersh et al. 2013). However, when compared to baseline pain, its duration of action is at least 2 h (Hersh et al. 2005). We expect based on impacted third molar surgery studies that rapid-release formulations of ibuprofen 400 mg would have an onset within 30 min and a duration of action of at least 4 to 6 h (Hersh et al. 2000; Theken et al. 2019). However, this assumption should be confirmed in placebo-controlled trials of toothache patients. Comparative studies are warranted to establish the place in therapy for each of these analgesic options.

Epidiolex, the formulation of CBD employed in the current study, is an FDA-approved prescription drug indicated for the treatment of seizures associated with Lennox-Gastaut syndrome, Dravet syndrome, or tuberous sclerosis complex in patients 1 y of age and older (US Food and Drug Administration 2018). It is pharmaceutical-grade CBD that has been extensively tested for efficacy, safety, and purity in patients with these rare seizure disorders (Hess et al. 2016; Devinsky et al. 2018; Miller et al. 2020). This is in contrast to the numerous CBD products available over-the-counter for which clinical research is sparse, claims are not closely scrutinized by the FDA, and product purity is often not accurately labeled (Miller et al. 2022; Spindle et al. 2022). Currently, Epidiolex is dispensed only by specialty pharmacies, so widespread use would be limited by availability as well as cost (>$1,000 for 3–7 doses). The use of Epidiolex to treat toothache pain in clinical practice would be considered “off-label use” because it is not approved to treat acute pain. While not prohibited by law, there should be a body of evidence that supports its use in the condition for which it is being prescribed (Verhagen et al. 2008). The data presented by Chrepa et al. (2023) suggest that CBD is efficacious for the treatment of toothache pain. However, a recent meta-analysis reported that CBD is ineffective in a variety of pain conditions (Moore et al. 2023). Thus, additional studies are necessary to establish whether CBD is a viable alternative analgesic for patients with toothache pain.

If further studies demonstrate efficacy, CBD could be used for relief of toothache pain as a “temporary bridge” until definitive dental care. However, even short-term use presents risks of adverse events and drug interactions. As demonstrated in the current publication, sedation, abdominal pain, and diarrhea do occur even with single-dose exposure, and patients must be counseled about the likelihood of these events. According to the package insert, the drug can raise liver enzymes and increase the risk of suicidal ideation (US Food and Drug Administration 2018). Thus, patients with preexisting liver dysfunction or a history of depression are poor candidates for CBD. CBD is an inhibitor of CYP1A2 and CYP2C9 with the potential to increase blood levels and associated toxicities of substrate drugs such as theophylline (arrhythmias and seizures), benzodiazepines (sedation and memory disturbances), and warfarin (bleeding) (US Food and Drug Administration 2018).

In conclusion, Chrepa et al. (2023) present promising results supporting a novel analgesic strategy for toothache pain. We look forward to future studies of CBD in acute dental pain seeking to establish efficacy and define its place in therapy.

## Author Contributions

K.N. Theken, E.V. Hersh, contributed to conception, design, data acquisition, analysis, and interpretation, drafted and critically revised the manuscript. All authors gave final approval and agree to be accountable for all aspects of the work.
